# Supplementary material for: Contextual factors predicting compliance behavior during the COVID-19 pandemic: A machine learning analysis on survey data from 16 countries
Source: PLoS One. 2022 Nov 28;17(11):e0276970. doi: 10.1371/journal.pone.0276970 (PMC9704675; doi:10.1371/journal.pone.0276970)

**Supporting information**

Contextual factors predicting compliance behavior during the Covid-19 pandemic: A machine learning analysis on survey data from 16 countries

Nandor Hajdu^1,2,*^, Kathleen Schmidt^3^, Gergely Acs^4^, Jan P. Röer^5^, Alberto Mirisola^6^, Isabella Giammusso^6^, Patrícia Arriaga^7^, Rafael Ribeiro^7^, Dmitrii Dubrov^8^, Dmitry Grigoryev^8^, Nwadiogo C. Arinze^9^, Martin Voracek^10^, Stefan Stieger^11^, Matus Adamkovic^12,13^, Mahmoud Elsherif^14^, Bettina M. J. Kern^10,15^, Krystian Barzykowski^176^, Ewa Ilczuk^176^, Marcel Martončik^12^, Ivan Ropovik^17,18^, Susana Ruiz-Fernandez ^19,20,21^, Gabriel Baník^12^, José Luis Ulloa^2221^, Balazs Aczel^2¶^, Barnabas Szaszi^2¶^

^1^Doctoral School of Psychology, ELTE Eötvös Loránd University, Budapest, Hungary

^2^Institute of Psychology, ELTE Eötvös Loránd University, Budapest, Hungary

^3^Southern Illinois University, Carbondale, Illinois, United States of America

^4^Department of Networked Systems and Services, Budapest University of Technology and Economics, Budapest, Hungary

^5^Department of Psychology and Psychotherapy, Witten/Herdecke University, Witten, Germany

^6^Department of Psychology, Educational Science and Human Movement, University of Palermo, Italy

^7^ISCTE-University Institute of Lisbon, CIS-IUL, Portugal

^8^National Research University Higher School of Economics, Russian Federation

^9^Alex Ekwueme Federal University, Ndufu-Alike, Nigeria

^10^Department of Cognition, Emotion, and Methods in Psychology, Faculty of Psychology, University of Vienna, Austria

^11^Department of Psychology and Psychodynamics, Division Psychological Methodology, Karl Landsteiner University of Health Sciences, Krems an der Donau, Austria

^12^Institute of Psychology, Faculty of Arts, University of Presov, Prešov, Slovakia

^13^Institute of Social Sciences, CSPS Slovak Academy of Sciences

^14^Department of Psychology, University of Birmingham, Birmingham, United Kingdom

^16^Department of European and Comparative Literature and Language Studies, Faculty of Philological and Cultural Studies, University of Vienna, Vienna, Austria

^17^Institute of Psychology, Faculty of Philosophy, Jagiellonian University, Krakow, Poland

^18^Faculty of Education, Charles University, Prague, Czech Republic

^19^Faculty of Education, University of Presov, Prešov, Slovakia

^20^FOM University of Applied Sciences, Essen, Germany

^21^Leibniz-Institut für Wissensmedien, Tübingen, Germany

^22^Programa de Investigación Asociativa (PIA) en Ciencias Cognitivas, Centro de Investigación en Ciencias Cognitivas (CICC), Facultad de Psicología, Universidad de Talca, Chile.

* Corresponding author Email: hajdu.nandor93@gmail.com (NH)

^¶^ BA and BS are Joint Senior Authors.

|  |
| --- |

**Deviations from Pre-registration**

While in our pre-registration we stated that we would train random forest models on Hungarian data only, we decided to use this method on data from every country for two reasons: the robustness of the method and the comparability of results. Our pre-registration also contained plans for cluster analyses, but we decided against performing them because they would not contribute to the identification of contextual factors that predict leaving home or the riskiness of the visited place - which was the main goal of the article.

**Supplementary Results**

**S1 Table 1. Data Collection Intervals Per Countries.**

The Stay at home recommendations or requirements column represents periods where governments either a.) recommended not leaving house, b.) required people not to leave house with exceptions for daily exercise, grocery shopping, and ‘essential’ trips, or c.) Required not to leave house with minimal exceptions (e.g. allowed to leave only once a week, or only one person can leave at a time), according to the Oxford Policy Tracker.

| **Country** | **Start date** | **Last participant** | **Stay at home recommendations or requirements** |
| --- | --- | --- | --- |
| Austria | 2020.04.29 | 2020.09.25 | 2020.03.16 - 04.30 |
| Germany | 2020.04.30 | 2020.11.12 | 2020.03.09 - 2020.05.05; |
| Greece | 2020.04.29 | 2020.06.26 | 2020.03.23 - 2020.09.28 |
| Hungary | 2020.04.29 | 2020.07.10 | 2020.03.13 - 2020.09.10 |
| Italy | 2020.05.01 | 2020.09.28 | 2020.02.23 - 2020.07.26 |
| Japan | 2020.05.01 | 2020.05.25 | 2020.04.07 - 2020.05.24 |
| Netherlands | 2020.04.29 | 2020.08.29 | 2020.03.06 - 2020.10.08 |
| Nigeria | 2020.05.31 | 2020.07.13 | 2020.03.23 - 2020.10.05. |
| Poland | 2020.04.30 | 2020.10.19 | 2020.03.31 - 2020.05.29 |
| Portugal | 2020.05.01 | 2020.11.10 | 2020.03.19 - 2020.08.24 |
| Romania | 2020.04.30 | 2020.05.30 | 2020.03.12 - 2020.10.08 |
| Russian Federation | 2020.05.01 | 2020.07.11 | 2020.03.05 - 2020.10.01 |
| Slovakia | 2020.05.01 | 2020.11.10 | 2020.03.12 - 2020.06.13 |
| Switzerland | 2020.05.01 | 2020.07.12 | 2020.03.17 - 2020.06.21 |
| United Kingdom | 2020.04.30 | 2020.09.04 | 2020.03.23 - 2020.10.06 |
| United States of America | 2020.04.29 | 2020.09.04 | 2020.03.15 - 2020.09.18 |

**S2 Figure 1. Data Collection Dates.**


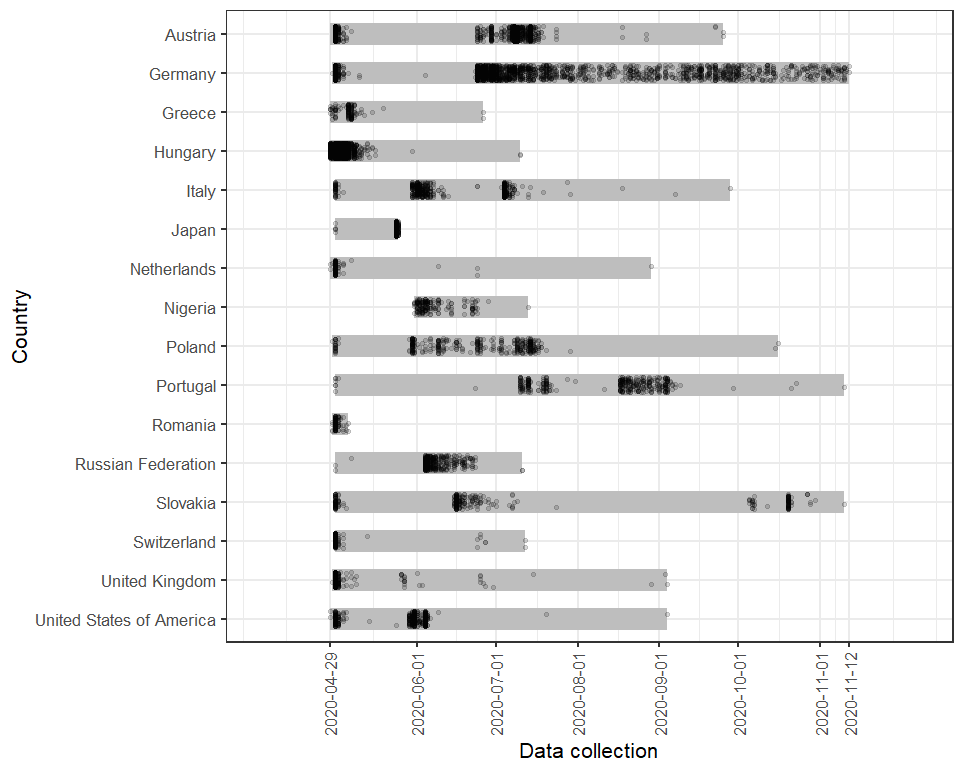

Supplement: S1 File — (DOCX) [file pone.0276970.s001.docx]
